# The Power Struggle: Kynurenine Pathway Enzyme Knockouts and Brain Mitochondrial Respiration

**DOI:** 10.1111/jnc.70075

**Published:** 2025-05-02

**Authors:** László Juhász, Krisztina Spisák, Boglárka Zsuzsa Szolnoki, Anna Nászai, Ágnes Szabó, Attila Rutai, Szabolcs Péter Tallósy, Andrea Szabó, József Toldi, Masaru Tanaka, Keiko Takeda, Kinuyo Ozaki, Hiromi Inoue, Sayo Yamamoto, Etsuro Ono, Mihály Boros, József Kaszaki, László Vécsei

**Affiliations:** ^1^ Institute of Surgical Research University of Szeged, Albert Szent‐Györgyi Medical School Szeged Hungary; ^2^ Department of Neurology University of Szeged, Albert Szent‐Györgyi Medical School Szeged Hungary; ^3^ HUN‐REN‐SZTE Neuroscience Research Group, Hungarian Research Network University of Szeged (HUN‐REN‐SZTE), Danube Neuroscience Research Laboratory Szeged Hungary; ^4^ Department of Physiology, Anatomy and Neuroscience University of Szeged Szeged Hungary; ^5^ Department of Biomedicine, Graduate School of Medical Sciences Kyushu University Fukuoka Japan; ^6^ Center of Biomedical Research, Research Center for Human Disease Modeling, Graduate School of Medical Sciences Kyushu University Fukuoka Japan

**Keywords:** kynurenic acid, kynurenine aminotransferase, mitochondrial dysfunction, neurodegenerative diseases, psychiatric diseases, transgenic mice, tryptophan

## Abstract

Numerous illnesses, including neurological and mental disorders, have been associated with mitochondrial dysfunction. Disruptions in mitochondrial respiration and energy production have been linked to dysmetabolism of the tryptophan (Trp)‐kynurenine (KYN) pathway, which produces a diverse array of bioactive metabolites. Kynurenic acid (KYNA) is a putative neuroprotectant. The exact mechanisms through which Trp‐KYN metabolic dysregulation affects mitochondrial function remain largely unclear. This study investigates the impact of the genetic deletion of kynurenine aminotransferase (KAT) enzymes, which are responsible for KYNA synthesis, on mitochondrial function, specifically mitochondrial respiration and ATP synthesis, and its potential role in neuropsychiatric pathology. CRISPR/Cas9‐induced knockout mouse strains kat1^−/−^, kat2^−/−^, and kat3^−/−^ were generated. Eight‐to‐ten‐week‐old male mice were used, and cerebral and hepatic respiration, complex I‐ and II‐linked oxidative phosphorylation (CI and CII OXPHOS), and complex IV (CIV) activity were measured using high‐resolution respirometry. Mitochondrial membrane potential changes were measured with Fluorescence‐Sensor Blue and safranin dye. KAT knockout mice exhibited significantly lower cerebellar respiration (CI OXPHOS, CII OXPHOS, and CIV activity) compared to wild‐type mice. Lower baseline respiration and attenuated OXPHOS activities were observed in the hippocampus and striatum, particularly in kat2^−/−^ and kat3^−/−^ mice. Non‐neuronal tissues showed reduced CIV activity, while ADP‐stimulated CI and CII OXPHOS remained unchanged. The deletion of the KAT genes significantly impairs mitochondrial respiration and ATP synthesis, potentially contributing to pathogenesis. This study highlights the importance of KYNA in mitochondrial function, offering new insights into potential therapeutic targets for various disorders. Targeting the KYN pathway could mitigate mitochondrial dysfunction in a variety of diseased conditions.

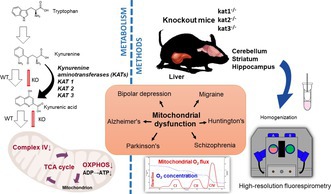

## Graphical abstract

Kynurenine pathway enzyme knockouts impair mitochondrial respiration. The deletion of kynurenine aminotransferase genes leads to significant impairment of mitochondrial respiration and ATP production, highlighting the critical role these enzymes play in cellular energy metabolism. Dysfunction in this pathway can contribute to a wide range of diseases, particularly those affecting the nervous system, such as neurological and psychiatric disorders. Targeting the kynurenine pathway represents a potential therapeutic strategy to mitigate mitochondrial dysfunction and restore cellular energy balance. CI, complex I; CII, complex II; CIV, complex IV; KO, knockout; OXPHOS, oxidative phosphorylation; TCA cycle, tricarboxylic acid (Krebs) cycle; WT, wild type.

**Abbreviations**
3‐HAO: 3‐hydroxyanthranilate oxidase
ACMSD: 2‐amino‐3‐carboxymuconic‐6‐semialdehyde decarboxylase
AHR: aryl hydrocarbon receptor
ATP: adenosine triphosphate
BNIP3: BCL2 interacting protein 3
Cas9: CRISPR‐associated protein 9
CCBL: cysteine conjugate beta‐lyase
CCCP: carbonyl cyanide m‐chlorophenyl hydrazone
CI and CII OXPHOS: complex I‐ and II‐linked oxidative phosphorylation
CIV: complex IV
CNS: central nervous system
CRISPR: clustered regularly interspaced short palindromic repeats
ETS: electron transport system
FADH_2_
: reduced form of flavin adenine dinucleotide
GPR35: G protein‐coupled receptor 35
hCG: human chorionic gonadotropin
IDOs: indoleamine 2,3‐dioxygenases
KAT KO: kynurenine aminotransferase knockout
KAT/KATs: kynurenine aminotransferase(s)
kat1^−/−^
: kynurenine aminotransferase I knockout mouse strain
kat2^−/−^
: kynurenine aminotransferase II knockout mouse strain
kat3^−/−^
: kynurenine aminotransferase III knockout mouse strain
KFA: kynurenine formamidase
KMO: kynurenine 3‐monooxygenase
KYN: kynurenine
KYNA: kynurenic acid
L‐KYN: L‐kynurenine
mPTP: mitochondrial permeability transition pore
NADH: reduced form of nicotinamide adenine dinucleotide
N‐f‐KYN: N‐formyl‐kynurenine
NMDA‐R: N‐methyl d‐aspartate receptor
PMSG: pregnant mare serum gonadotropin
QPRT: quinolinate phosphoribosyl transferase
Saf: safranin
TCA: tricarboxylic cycle acid
TDO: tryptophan‐2,3‐deoxygenase
TMPD: N,N,N′,N′‐tetramethyl‐*p*‐phenylenediamine dihydrochloride
Trp: tryptophan
wt: wild‐type
ΔΨmt: mitochondrial membrane potential

## 1 Introduction

Mitochondrial dysfunction has emerged as a critical factor in the development of various neurological and psychiatric disorders. Mitochondria, the powerhouse of the cell, are essential for energy production, cellular metabolism, and the regulation of apoptotic pathways (Martin 2010; Bustamante‐Barrientos et al. 2023). Disruptions in mitochondrial function can lead to an imbalance in cellular energy homeostasis, contributing to the pathogenesis of diseases such as Alzheimer's, Parkinson's, and schizophrenia (Golpich et al. 2017; Norat et al. 2020). Additionally, the intricate relationship between mitochondrial activity and oxidative stress underscores the importance of maintaining mitochondrial health for overall neurological function (Carrì et al. 2015; Song et al. 2021). Understanding the underlying mechanisms that influence mitochondrial efficiency is critical for developing effective therapeutic strategies for these debilitating conditions.

The tryptophan (Trp)‐kynurenine (KYN) metabolic pathway is currently being extensively studied as a potential new target for intervention, due to its crucial involvement in various neurological conditions (Fujigaki et al. 2017; Mithaiwala et al. 2021). This pathway plays a crucial role in both fundamental research and experimental treatments because it has a substantial influence on the development of these disorders. These pathologies include various neurodegenerative diseases, for example, Alzheimer's, Huntington's, Parkinson's, and psychiatric diseases associated with mood disorders, such as bipolar depression and migraine (Han et al. 2010; Vécsei et al. 2013; Sas et al. 2018; Tanaka et al. 2020). The Trp‐KYN pathway is considered the primary pathway of Trp amino acid metabolism; nearly 95% of Trp is converted via this route (Tanaka et al. 2021, 2022; Savitz 2020).

**FIGURE 1:**
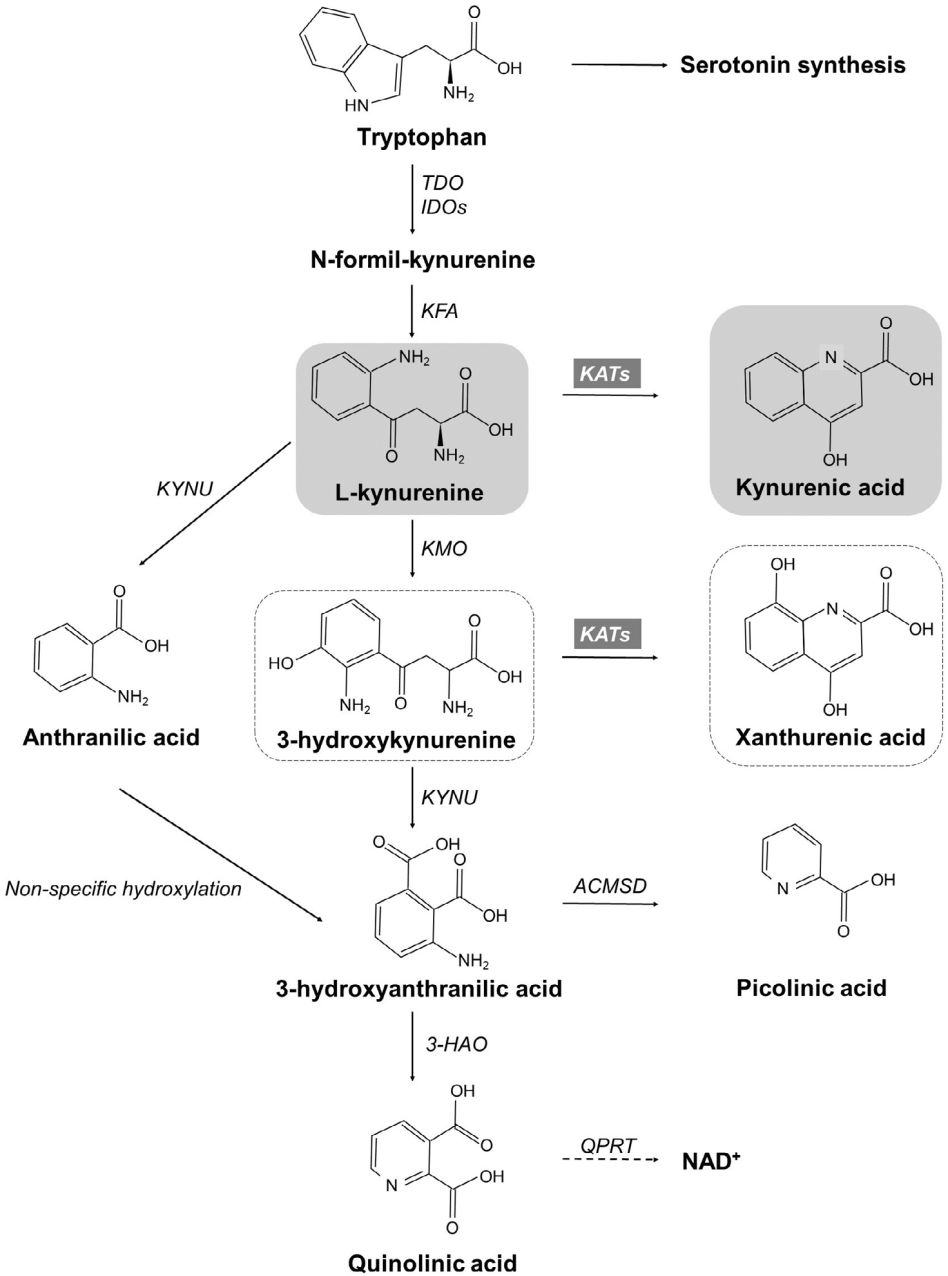
Enzymatic conversion of tryptophan and L‐kynurenine to kynurenic acid. Tryptophan is oxidized to N‐formyl‐kynurenine, which is catalyzed by tryptophan 2,3‐dioxygenase (TDO) or the less specific indoleamine 2,3‐dioxygenases (IDOs). N‐formyl‐kynurenine is then hydrolyzed to L‐kynurenine (L‐KYN) by kynurenine formamidase (KFA). The irreversible conversion of L‐KYN to kynurenic acid (KYNA) is catalyzed by kynurenine aminotransferase isozymes (KATs; indicated with gray box). 3‐HAO, 3‐hydroxyanthranilate oxidase; ACMSD, 2‐amino‐3‐carboxymuconic‐6‐semialdehyde decarboxylase; IDOs, indoleamine 2,3‐dioxygenases; KATs, kynurenine aminotransferases; KFA, kynurenine formamidase; KMO, kynurenine 3‐monooxygenase; KYNU, kynureninase; QPRT, quinolinate phosphoribosyl transferase;TDO, tryptophan 2,3‐dioxygenase.

At the beginning of the reaction sequence (Figure 1), Trp is oxidized to N‐formyl‐KYN (N‐f‐KYN), which is catalyzed by tryptophan 2,3‐dioxygenase or the less specific indoleamine 2,3‐dioxygenases. N‐f‐KYN is then hydrolyzed to L‐kynurenine (L‐KYN) by kynurenine formamidase. The irreversible conversion of L‐KYN to kynurenic acid (KYNA) is catalyzed by members of the pyridoxal‐5'‐phosphate‐dependent enzymes, kynurenine aminotransferase isozymes (KATs or KAT I‐IV).

Two Trp by‐products of this pathway, L‐KYN and KYNA, have distinct physiological effects in the central nervous system (CNS) and non‐neuronal tissues. While the L‐KYN metabolite, quinolinic acid, is an N‐methyl d‐aspartate receptor (NMDA‐R) agonist and is implicated in neurotoxicity, mitochondrial dysfunction, neurodegenerative disease, and psychiatric disorders, KYNA is an endogenous NMDA‐R inhibitor and plays an important role in immunosuppression, cytoprotection, vasodilation, and the maintenance of mitochondrial and intestinal homeostasis (Wirthgen et al. 2018; Seo and Kwon 2023; Varga et al. 2010; Érces et al. 2012).

Elevated L‐KYN levels and/or alterations in the L‐KYN/KYNA ratio, along with other KYN pathway metabolites (e.g., anthranilic acid, xanthurenic acid, 3‐hydroxykynurenine, and 3‐hydroxyanthranilic acid), play a significant role in the development of mitochondrial dysfunction, including impairments in mitochondrial oxidative phosphorylation (Baran et al. 2016; Palzkill et al. 2022).

Therefore, maintaining the physiological L‐KYN/KYNA ratio and endogenous KYNA levels may have therapeutic potential in certain diseases (Johansson et al. 2013; Liu et al. 2018).

So far, four KAT enzymes have been identified in the mammalian brain. These include KAT I, also referred to as glutamine transaminase K/cysteine conjugate beta‐lyase (CCBL) 1, KAT II, which is aminoadipate aminotransferase, KAT III, known as glutamine transaminase L/CCBL 2, and KAT IV, which is glutamic‐oxaloacetic transaminase 2 or mitochondrial aspartate aminotransferase (Han et al. 2010; Tanaka et al. 2021). Of these enzymes, KAT II is considered the key enzyme responsible for KYNA production in the mammalian brain (Tanaka et al. 2021).

In addition to L‐KYN‐KYNA conversion, KATs have a broad substrate specificity and function; all four isozymes are involved in reactions linked to the Szent‐Györgyi‐Krebs cycle (tricarboxylic acid cycle [TCA]) taking place in the mitochondrion (Tanaka et al. 2022).

For instance, cytosolic KAT I catalyzes the conversion of an S‐substituted l‐cysteine to pyruvate and also facilitates the conversion of L‐glutamine to α‐ketoglutarate. KAT II and KAT III catalyze the transformation of α‐ketoglutarate into L‐glutamate. Additionally, KAT II catalyzes the conversion of α‐ketoglutarate to 2‐oxoadipate, which is ultimately broken down into acetyl‐CoA. KAT III also catalyzes the conversion of an S‐substituted l‐cysteine to pyruvate.

Although exogenous KYNA and its synthetic analogues (SZR‐72 and SZR‐104)‐mediated mitoprotective effects are well‐characterized in neuronal and non‐neural tissues under inflammatory and ischemic–hypoxic conditions (Juhász et al. 2020; Poles et al. 2021; Balla et al. 2021), little information is available on the mitochondrial effects of endogenous KYNA and the role of synthesizing KAT enzymes in mitochondrial metabolism.

Since the majority of KYNA is synthesized by the mitochondrial KAT II in the CNS (encoded by aadat gene), it can be assumed that the regulatory function of endogenous KYNA can be detected by the in vivo manipulation of the isozyme. Our hypothesis is that endogenous KYNA acts as a regulator of the mitochondrial electron transport and ATP synthesis, and KATs modulation may serve as a therapeutic target for preclinical research. Given this background, the objective of the present study was to investigate the mitochondrial effects of KYNA‐producing KAT enzymes using newly created kynurenine aminotransferase I, II, and III enzyme‐deficient mouse strains (kat1^−/−^, kat2^−/−^, and kat3^−/−^; Szabó et al. 2025). More directly, our aim was to compare cerebral mitochondrial O_2_ consumption in different brain regions of KAT knockout (KO) animals and find a connection between mitochondrial respiration and mitochondrial membrane potential (ΔΨmt).

## 2 Materials and Methods

Three KAT KO strains (kat1^−/−^, kat2^−/−^, and kat3^−/−^) were generated using the CRISPR/Cas9 method on C57BL/6NCrSlc mice (Szabó et al. 2025) and used in our studies. High‐Resolution FluoRespirometry was applied to determine mitochondrial O_2_ flux or ΔΨmt from brain (cerebellum, hippocampus, striatum) and liver tissue homogenates.

### 2.1 CRISPR/Cas9 Genome Editing

To generate KAT KO mice using the CRISPR/Cas9 method, C57BL/6NCrSlc and Crl:CD1 mice were purchased from Japan SLC Inc. (Hamamatsu, Japan) and Charles River Laboratories International Inc. (Yokohama, Japan), respectively. Animals were kept in plastic cages (four to five per cage) under pathogen‐free conditions (sentinel mice were examined periodically throughout the study) in a room maintained at 23°C±3°C and 50%±15% relative humidity under a 12:12‐h light:dark cycle. Mice had free access to commercial chow (CRF‐1LID10; Oriental Yeast, Tokyo, Japan) and water throughout the study. Animal experiments were carried out humanely in accordance with the Regulations for Animal Experiments of Kyushu University, and Fundamental Guidelines for Proper Conduct of Animal Experiment and Related Activities in Academic Research Institutions under the jurisdiction of the Ministry of Education, Culture, Sports, Science, and Technology of Japan and with approval from the Institutional Animal Experiment Committees of Kyushu University (the ethics approval reference number: A29‐338‐1).

The CRISPR/Cas9 technique was used to introduce a deletion in the KATs genes. The sgRNAs were selected using CRISPRdirect software (Naito et al. 2015). The artificially synthesized sgRNAs were purchased from FASMAC Co. Ltd. (Atsugi, Japan). Female C57BL/6NCrSlc mice were injected with 5 U/0.2 mL of pregnant mare serum gonadotropin (SEROTOROPIN, ASKA Animal Health Co. Ltd., #2210‐05, Tokyo, Japan), and 5 U/0.2 mL of human chorionic gonadotropin (GONATROPIN, ASKA Pharmaceutical Co. Ltd., #123‐00078‐9, Tokyo, Japan), administered 48 h apart, and then mated with male C57BL/6NCrSlc mice.

The superovulating female mice were sacrificed by cervical dislocation. The mice were then dissected to expose the abdominal cavity, and the uteruses, oviducts, and ovaries were removed.

Fertilized one‐cell embryos were collected from the oviducts. Subsequently, 25 ng/μL of sgRNA and 75 ng/μL of Guide‐it Recombinant Cas9 protein (TaKaRa Bio Inc., #632641, Kusatsu, Japan) were injected into the pronuclei of one‐cell‐stage embryos (Table 1). These one‐cell‐stage embryos were cultured in M2 medium (Sigma‐Aldrich, #7167, St. Louis, MO, USA) overnight in a CO_2_ incubator maintained at 5% CO_2_ concentration at 37°C. The next day, the injected two‐cell embryos were then transferred into pseudopregnant Crl:CD1 mice prepared by mating with vasectomized Crl:CD1 male mice. Briefly, female mice on Day 1 of pseudopregnancy were anesthetized with a combination anesthetic (Kawai et al. 2011) prepared with 0.3 mg/kg of medetomidine (DOBERNE, Nippon Zenyaku Kogyo Co. Ltd., #10008816, Koriyama, Japan); 4 mg/kg of midazolam (DORMICUM, Maruishi Pharmaceutical Co. Ltd., #211‐762 100, Osaka, Japan); and 5 mg/kg of butorphanol (VETORPHALE, Meiji Animal Health Co. Ltd., #551‐04005‐1, Kumamoto, Japan) by intraperitoneal injection.

**TABLE 1:** sgRNA for genome‐editing.

Name	Sequence
M‐KAT I‐2	GCTTAGGAGCAGGACTCTGCGguuuuagagcuagaaauagcaaguu
	aaaauaaggcuaguccguuaucaacuugaaaaaguggcacggacucggugcuuuu
M‐KAT II‐2	GTTCCTCACTGCAACGAGCCguuuuagagcuagaaauagcaaguu
	aaaauaaggcuaguccguuaucaacuugaaaaaguggcacggacucggugcuuuu
M‐KAT III‐2‐4	GCTTGTGGCCGTGGGGGCATAguuuuagagcuagaaauagcaaguu
	aaaauaaggcuaguccguuaucaacuugaaaaaguggcacggacucggugcuuuu

The ovary, oviduct, and part of the uterine horn were pulled out through an incision made in the rear back of the female mouse. Embryo transfer into the oviduct was carried out by inserting a capillary into the infundibulum and expelling embryos toward the ampulla. The capillary was then gently withdrawn from the infundibulum. The ovary, oviduct, and uterine horn were pushed back into the abdomen, and the wound was closed using surgical sutures. The mice were kept warm on a warming plate at 37°C until recovered from the effects of anesthesia.

### 2.2 DNA Extraction and Sequencing

Genomic DNA was extracted from the tails of mice using the NucleoSpin Tissue kit (MACHEREY‐NAGEL GmbH & Co, #740952.10, KG, Düren, Germany). Each targeted fragment surrounding the sgRNA binding site within the KAT genes was amplified from the extracted genomic DNA using TAKARA Ex Taq (TaKaRa Bio Inc., #RR001A, Kusatsu, Japan) and the first pair of primers, followed by amplification with the second pair of primers (Table 2). The primers were purchased from Medical & Biological Laboratories Co. Ltd. (Tokyo Japan). The PCR product was purified using the Fast Gene Gel/PCR Extraction Kit (Nippon Genetics Co. Ltd., #FG‐91202, Tokyo, Japan), and further purified by agarose gel electrophoresis and the Monarch DNA Gel Extraction Kit (NEW ENGLAND BioLabs Inc., #T1020, Ipswich, MA, USA). Finally, the PCR products were sequenced using one of the second primers.

**TABLE 2:** Primers for PCR and sequencing.

Name of primer	Sequence
M‐KAT I‐2_1st_F	TGAGGGACCCAAGGTATGAG
M‐KAT I‐2_1st_R	TGGATCCCACTGCATAGACA
M‐KAT I‐2_2nd_F	ACCCAAGGTATGAGCTGAGC
M‐KAT I‐2_2nd_R^a^	GCATAGACAGATAAAGTCAC
M‐KAT II_1st_F	CCCTCTGTGGATGGACTTTG
M‐KAT II_1st _R	TTGAAAGATGTGCCTCATGC
M‐KAT II_2nd_F	GGATGGACTTTGTCCCTTCT
M‐KAT II_2nd_R^a^	ATGTGCCTCATGCTTGGCCC
M‐KAT III‐2_1st_F	TCCACTCATTTGGTGCACAT
M‐KAT III‐2_1st_R	TCTTTGTGTGTGCGATGGTT
M‐KAT III‐2_2nd_F^a^	TGGTGCACATACACATGTGT
M‐KAT III‐2_2nd_R	GTGCGATGGTTACATGGTGG

^a^ Primer for sequencing.

### 2.3 Generation of KAT KO Mice by the CRISPR/Cas9 Method

To create KO mice for the KAT genes, 25 ng/μL of each sgRNA and 75 ng/μL of Cas9 protein were injected into the pronuclei of one‐cell‐stage embryos. Sequencing analyses of the founder mice revealed various deletions and/or insertions in each target sequence (Figures S1 and S2). From each KAT gene, one founder was selected to establish a homozygous mouse line for further analysis (Table 3). KAT I and KAT III KO mouse lines express a carboxy‐terminal truncated polypeptide consisting of the first 149 or 150 amino acids of the intact KAT I or KAT III with 2 or 7 nucleotide deletions (CCDS nucleotide sequence 439‐440 or 404–410) in the mRNA, respectively. KAT II KO mouse line was generated as previously described (Szabó et al. 2025). KAT II KO mouse line expresses a carboxy‐terminal truncated polypeptide that includes only the first 47 amino acids of the intact KAT II with 2 nucleotide deletions (CCDS nucleotide sequence 32‐33) in the mRNA, although there is a possibility that the ribosome takes the second ATG in the same reading frame and releases an amino‐terminal truncated polypeptide consisting of 381 amino acids of KAT II.

**TABLE 3:** Nucleotide sequences of KAT genes.

Name	Transcript id	CCDS	CCDS nucleotide sequence
Kyat1‐204	ENSMUST00000113661.10	CCDS15874	439‐440 (2 n.t. deletion)
Aadat‐201	ENSMUST00000079472.4	CCDS22320	32‐33 (2 n.t. deletion)
Kyat3‐201	ENSMUST00000044392.11	CCDS17881	404‐410 (7 n.t. deletion)
Got2‐201	ENSMUST00000034097.8	CCDS22568	179‐180 (A insertion)

### 2.4 Experimental Animals

The experiments were performed on 8‐10‐week‐old, male C57BL/6N wild‐type (wt), kat1^−/−^, kat2^−/−^, and kat3^−/−^ mice. A total of 42 animals (*n* = 42) were used, with 7 animals per group. After anesthesia with a mixture of ketamine and xylazine (80 mg/kg and 24 mg/kg, *ip*), liver samples from the left lateral lobe were taken, and the animals were decapitated for the removal of the cerebellum, striatum, and hippocampus. Maintenance of mouse colonies was carried out by our collaboration partner Neuroscience Research Group, Hungarian Academy of Sciences (MTA)‐University of Szeged (SZTE) (Szabó et al. 2025; Animal license no: XI./95/2020).

### 2.5 Sample Size Determination

Sample size was estimated using ClinCalc sample size calculator (https://clincalc.com/stats/samplesize.aspx) assuming a 15% change (increase or decrease) in OXPHOS capacity. Given a presumed true hazard ratio of 0.2, with a statistical power of 0.8 (1−*β*) and a Type I error of *α* =0.05, it was recommended to include seven wild‐type and seven KAT KO mice for respirometry analysis.

### 2.6 Assessment of Mitochondrial Respiration

**FIGURE 2:**
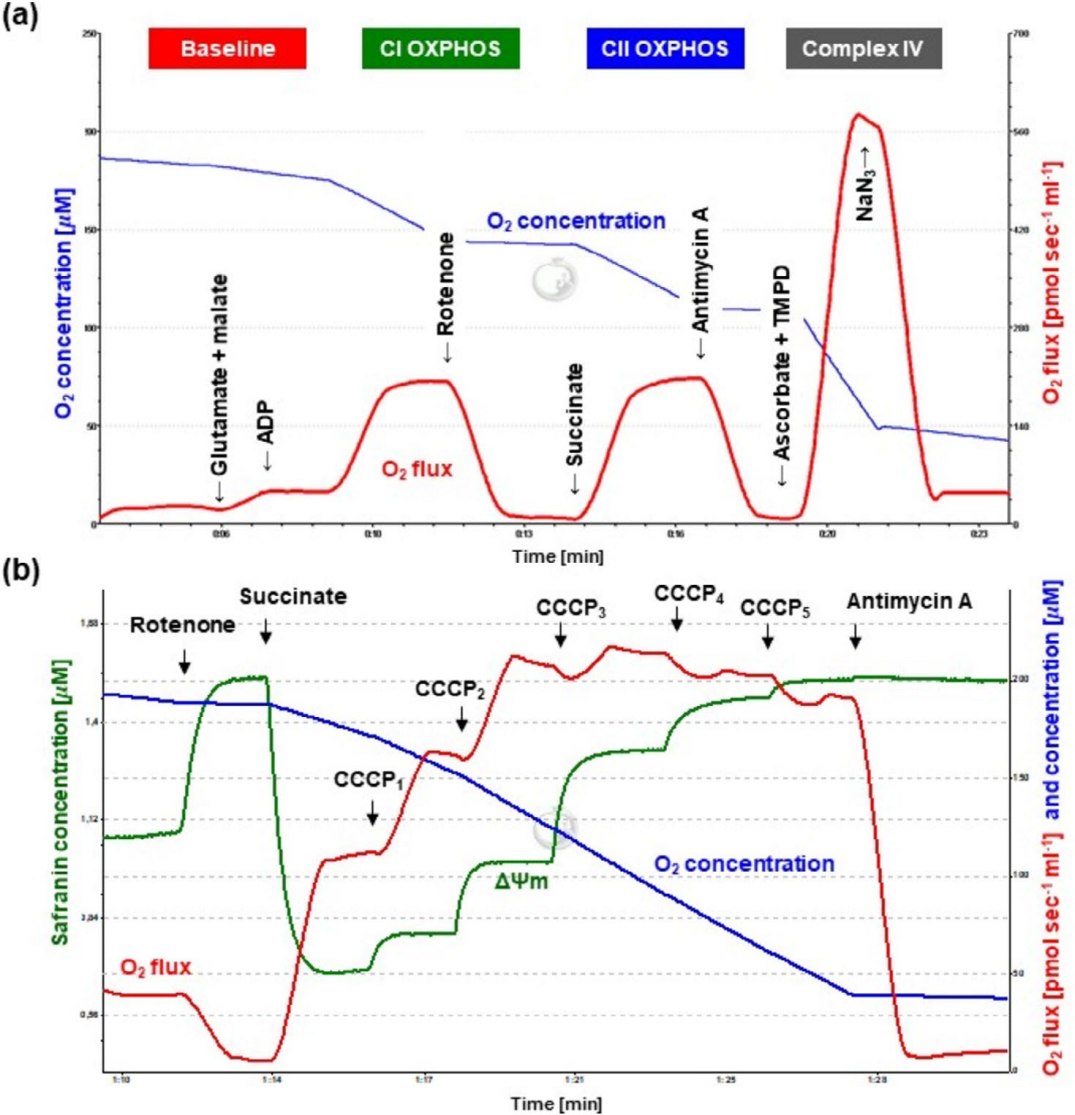
Protocols for the assessment of mitochondrial respiration and membrane potential (ΔΨmt). Baseline and complex‐specific respiratory activities were assessed in cerebellum, striatum, hippocampus and liver homogenates (a). Complex I was activated by glutamate and malate while, Complex II was activated by succinate in the presence of ADP (CI and CII OXPHOS). Complex IV activity was measured after the addition of ascorbate/TMPD. After stable steady‐state respiration, Complexes I, II, and IV were blocked with specific inhibitors (rotenone, antimycin A, and NaN_3_). Superimposed lines illustrate mitochondrial O_2_ consumption (O_2_ flux; red) and chamber O_2_ concentration (blue). Changes in cerebellar ΔΨmt following substrate (succinate), uncoupler (CCCP), and inhibitor (antimycin A) titration (b). Superimposed lines illustrate ΔΨmt (green), mitochondrial O_2_ consumption (O_2_ flux; red), and O_2_ concentration (blue). ADP, adenosine diphosphate; NaN_3_, sodium azide.

Mitochondrial oxygen consumption (O_2_ flux; red line; Figure 2) was assessed in three different brain regions (cerebellum, striatum and hippocampus; 5% w/v) and liver homogenates (10% w/v) using High‐Resolution FluoRespirometry (Oxygraph‐2k, Oroboros Instruments, Innsbruck, Austria; Juhász et al. 2020; Poles et al. 2021). All samples were cut with sharp scissors in Mir05 medium, and homogenates were prepared using a Potter–Elvehjem tissue grinder. Briefly, Complex I‐linked oxidative phosphorylation (CI OXPHOS) was measured in the presence of complex I‐linked substrates (10 mM glutamate and 2 mM malate; Sigma‐Aldrich, #G1626 and Sigma‐Aldrich, #M1000) and ADP (2.5 mM; Sigma‐Aldrich, #A5285). Rotenone (0.5 μM; Sigma‐Aldrich, #R8875) was used to (a) inhibit Complex I and (b) evaluate Complex II‐dependent oxidative phosphorylation (CII OXPHOS) in the presence of succinate (10 mM; Sigma‐Aldrich, #S2378) and adenylate. After inhibition of complex III (antimycin A; 2.5 μM; Sigma‐Aldrich, #A8674), Complex IV (CIV) respiratory activity was measured with ascorbate (2 mM; Sigma‐Aldrich, #A7631) and artificial substrate TMPD (0.5 mM; Sigma‐Aldrich, #T3134). Ascorbate was administered before TMPD to avoid uncontrollable autoxidation of the electron donor. Sodium azide (NaN_3_; 100 mM; Sigma‐Aldrich, #S2002) was finally added to block CIV‐linked mitochondrial O_2_ consumption. All the measurements were performed in a Mir05 respiration medium under continuous magnetic stirring (750 rpm) at 37°C. The DatLab software (version 7.4.0.4.; Oroboros Instruments, Innsbruck, Austria) was used for online display, respirometry data acquisition, and analysis.

### 2.7 Mitochondrial Membrane Potential (ΔΨmt)

The kat2^−/−^ mouse strain was selected for ΔΨmt measurements, since endogenous KYNA is mainly produced by KAT II in the brain tissues and KAT II is exclusively located in the mitochondria. Changes in ΔΨmt were assessed in cerebellum homogenate (5% w/v) with a cationic fluorescent probe, safranin (Saf; Sigma‐Aldrich, # S2255). The probe accumulates in energized mitochondria according to the inside negative potential with a concomitant change in absorption and fluorescence (Krumschnabel et al. 2014). Then, 2 μM Saf concentration does not affect complex II‐linked coupled respiration and fluorescence spectral changes are linearly related to ΔΨmt within a 0.5–2 μM concentration range (Juhász et al. 2020). The Fluorescence Sensor Blue (Oroboros Instruments, Austria) equipped with a filter set for safranin (excitation 465 nm; gain for sensor: 1000; polarization voltage: 500 mV) was connected through the front windows of the glass chambers to the fluorescence control unit for the measurement of Saf fluorescence. The respiration medium, stirring speed, and chamber temperature in these experiments were identical to those previously described above. Black cover slips were used to prevent light penetrating through the capillary and to avoid disruption of the fluorescence signal. Cerebellar samples were energized with succinate (10 mM; Sigma‐Aldrich, #S2378) after inhibition of Complex I with rotenone (0.5 μM; Sigma‐Aldrich, #R8875). After stabilization of respiration and fluorescence, a protonophorous uncoupler, carbonyl cyanide m‐chlorophenyl hydrazone (CCCP; Sigma‐Aldrich, #C2759), was titrated into a respiration chamber (0.05 μM in steps) for the stepwise depolarization of ΔΨmt. When ΔΨmt was collapsed after optimum concentration of CCCP, uncoupled respiration was inhibited with antimycin A (2.5 μM; Sigma‐Aldrich, #A8674). Changes in Saf fluorescence, an indicator of ΔΨmt, were expressed in μM, based on a standard calibration curve (Figure 2).

### 2.8 Statistical Analysis

All data were analyzed and illustrated without performing formal statistical tests for outliers, and no data points were excluded from the analysis. Data analysis was performed using a statistical software package (SigmaStat 13.0 for Windows; Jandel Scientific, Erkrath, Germany). Normality of data distribution was analyzed with the Shapiro–Wilk test. Differences in O_2_ flux between wt and KAT KO groups were calculated by one‐way analysis of variance (ANOVA) followed by the Holm–Sidak post hoc test. Differences in cerebellar ΔΨmt between wt and kat2^−/−^ groups were compared using Student's *t* test. Median values and 75th and 25th percentiles are provided in the figures. A value of *p* < 0.05 was considered statistically significant.

## 3 Results

### 3.1 Changes in Mitochondrial O_2_ Consumption in the Cerebellum

**FIGURE 3:**
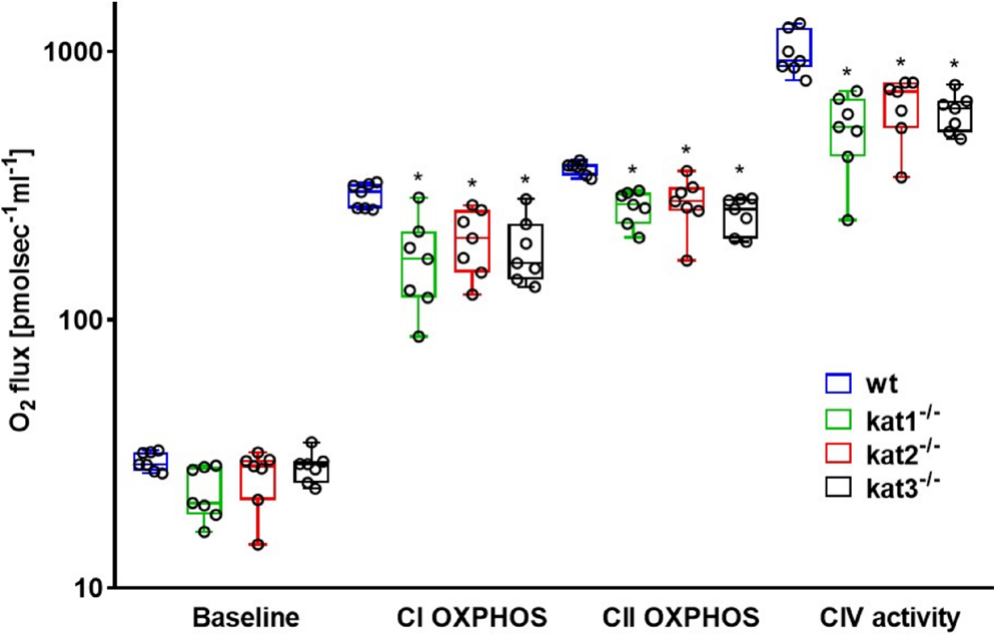
Baseline respiration, complex I‐, and complex II‐linked maximal capacities of oxidative phosphorylation (CI and CII OXPHOS) and complex IV (CIV) activities in wt (blue box), kat1^−/−^ (green box), kat2^−/−^ (red box), and kat3^−/−^ (black box) mouse cerebellum (*n* = 7 homogenized cerebellum samples from seven mice in each group). The plots illustrate the median (horizontal line in the box) and the 25th (lower whisker) and 75th (upper whisker) percentiles. One‐way ANOVA with Holm–Sidak post hoc tests was conducted to determine the differences between the wt and KAT KO mouse strains. *p < 0.05 versus wt group.

Baseline respiration registered after chamber closure without external substrate was not markedly different in wt and KAT KO cerebellar samples (*n* = 7, df_1_ = 3, df_2_ = 24, *F* = 2.928, *p* = 0.054, one‐way ANOVA). The addition of complex‐specific substrates into the oxygraph chambers increased mitochondrial oxygen consumption rates in all experimental groups, which were effectively blocked by ETS inhibitors (at complex I and III) to a value close to 0 pmol × sec^−1^ × mL^−1^ (Figure 2a). Compared to the wt group, kat1^−/−^, kat2^−/−^, and kat3^−/−^ animals showed lower O_2_ flux values in all the key parameters of mitochondrial respiration. Thus, complex I‐linked oxidative phosphorylation (CI OXPHOS; *n* = 7, df_1_ = 3, df_2_ = 24, *F* = 7.458, *p* (kat1^−/−^) = 0.002, *p* (kat2^−/−^) = 0.015, *p* (kat3^−/−^) = 0.005, one‐way ANOVA), complex II‐linked oxidative phosphorylation (CII OXPHOS; *n* = 7, df_1_ = 3, df_2_ = 24, *F* = 11.929, *p* (kat1^−/−^) < 0.001, *p* (kat2^−/−^) = 0.001, *p* (kat3^−/−^) < 0.001, one‐way ANOVA), and CIV‐dependent activities (*n* = 7, df_1_ = 3, df_2_ = 24, *F* = 13.038, *p* (kat1^−/−^) < 0.001, *p* (kat2^−/−^) < 0.001, *p* (kat3^−/−^) < 0.001, one‐way ANOVA) were significantly decreased in all three KO strains (Figure 3).

### 3.2 Changes in Mitochondrial O_2_ Consumption in the Hippocampus

**FIGURE 4:**
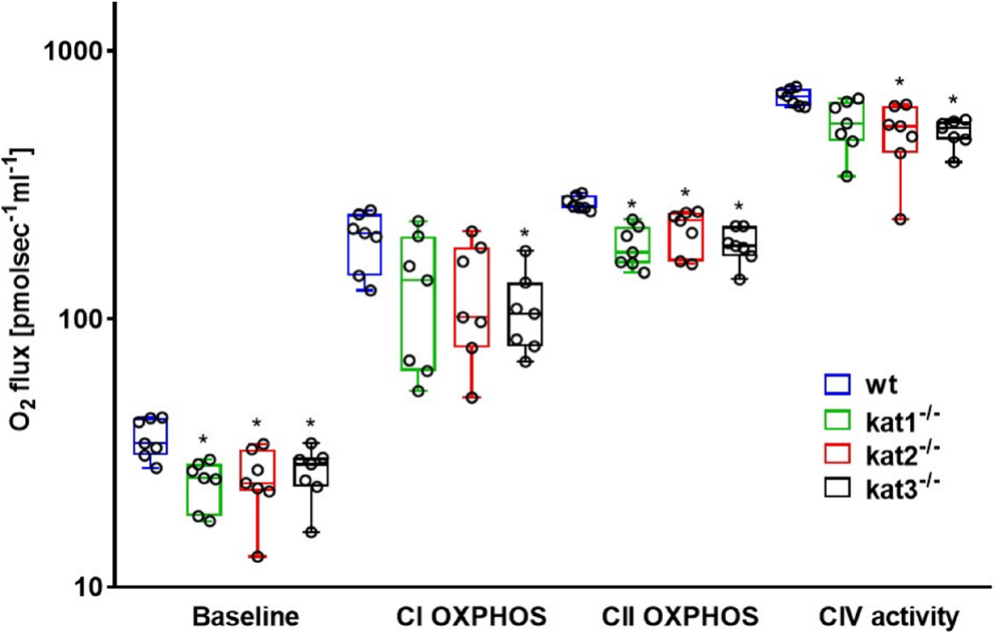
Baseline respiration, complex I‐, and complex II‐linked maximal capacities of oxidative phosphorylation (CI and CII OXPHOS) and complex IV (CIV) activities in wt (blue box), kat1^−/−^ (green box), kat2^−/−^ (red box), and kat3^−/−^ (black box) mouse hippocampus (*n* = 7 homogenized hippocampus samples from seven mice in each group). The plots demonstrate the median (horizontal line in the box) and the 25th (lower whisker) and 75th (upper whisker) percentiles. One‐way ANOVA with Holm–Sidak post hoc tests was conducted to determine the differences between the wt and KAT KO mouse strains. *p < 0.05 versus wt mice.

Figure 4 illustrates hippocampal respiration rates registered in wt and KAT KO animals. Compared to wt mice, all three KAT enzyme‐deficient strains exhibited significantly lower mitochondrial baseline respiration (*n* = 7, df_1_ = 3, df_2_ = 24, *F* = 5.482, *p* (kat1^−/−^) = 0.009, *p* (kat2^−/−^) = 0.014, *p* (kat3^−/−^) = 0.033, one‐way ANOVA) and CII OXPHOS capacity (*n* = 7, df_1_ = 3, df_2_ = 24, *F* = 11.612, *p* (kat1^−/−^) < 0.001, *p* (kat2^−/−^) = 0.009, *p* (kat3^−/−^) < 0.001, one‐way ANOVA). In addition, CIV activities were markedly reduced in kat2^−/−^ (*n* = 7, df_1_ = 3, df_2_ = 24, *F* = 5.371, *p* = 0.011, one‐way ANOVA) and kat3^−/−^ (*n* = 7, df_1_ = 3, df_2_ = 24, *F* = 5.371, *p* = 0.012, one‐way ANOVA) mice, and a decreasing tendency was observed in the kat1^−/−^ strain (*n* = 7, df_1_ = 3, df_2_ = 24, *F* = 5.371, *p* = 0.056, one‐way ANOVA). Although complex I‐supported respiration exhibited very low O_2_ consumption values in nearly half of the mice (kat1^−/−^: *n* = 3; kat2^−/−^: *n* = 3; kat3^−/−^: *n* = 3), this parameter was statistically significant in kat3^−/−^ (CI OXPHOS; *n* = 7, df_1_ = 3, df_2_ = 24, *F* = 3.616, *p* = 0.032, one‐way ANOVA) but not in kat1^−/−^ (*n* = 7, df_1_ = 3, df_2_ = 24, *F* = 3.616, *p* = 0.115, one‐way ANOVA) and kat2^−/−^ mice (*n* = 7, df_1_ = 3, df_2_ = 24, *F* = 3.616, *p* = 0.103, one‐way ANOVA).

### 3.3 Changes in Mitochondrial O_2_ Consumption in the Striatum

**FIGURE 5:**
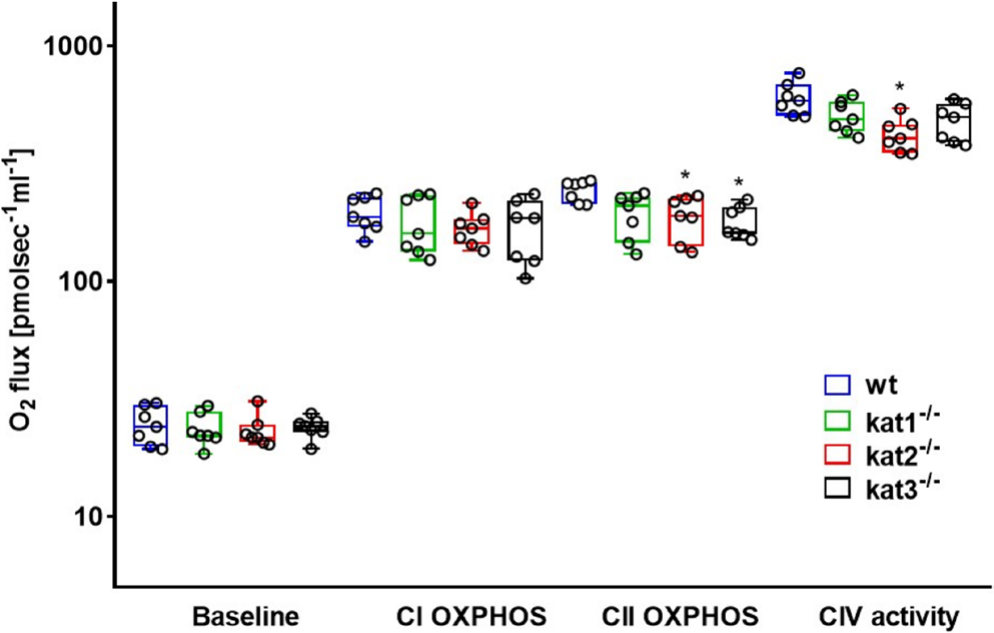
Baseline respiration, complex I‐, and complex II‐linked maximal capacities of oxidative phosphorylation (CI and CII OXPHOS) and complex IV (CIV) activities in wt (blue box), kat1^−/−^ (green box), kat2^−/−^ (red box), and kat3^−/−^ (black box) mouse striatum (*n* = 7 homogenized striatum samples from seven mice in each group). The plots demonstrate the median (horizontal line in the box) and the 25th (lower whisker) and 75th (upper whisker) percentiles. One‐way ANOVA with Holm–Sidak post hoc tests was conducted to determine the differences between the wt and KAT KO mouse strains. *p < 0.05 versus wt mice.

The KAT KO‐induced changes in striatal ETS function are illustrated in Figure 5. In comparison with wt mice, both kat2^−/−^ and kat3^−/−^ resulted in a significant decrease in CII‐dependent OXPHOS (*n* = 7, df_1_ = 3, df_2_ = 24, *F* = 4.739, *p* (kat2^−/−^) = 0.037, *p* (kat3^−/−^) = 0.013, one‐way ANOVA), and a decreasing tendency in this parameter was present in kat1^−/−^ animals as well (*n* = 7, df_1_ = 3, df_2_ = 24, *F* = 4.739, *p* = 0.052, one‐way ANOVA). As a result of kat2^−/−^, CIV activity was dropped markedly (*n* = 7, df_1_ = 3, df_2_ = 24, *F* = 5.615, *p* = 0.003, one‐way ANOVA), and a decreasing trend in striatal CIV activity was present in the kat3^−/−^ group (*n* = 7, df_1_ = 3, df_2_ = 24, *F* = 5.615, *p* = 0.056, one‐way ANOVA). There was no considerable difference in baseline respiration (*n* = 7, df_1_ = 3, df_2_ = 24, *F* = 0.184, *p* = 0.906, one‐way ANOVA) and CI‐linked OXPHOS (*n* = 7, df_1_ = 3, df_2_ = 24, *F* = 0.676, *p* = 0.575, one‐way ANOVA) between wt and KAT KO animals.

### 3.4 Changes in Mitochondrial O_2_ Consumption in the Liver

**FIGURE 6:**
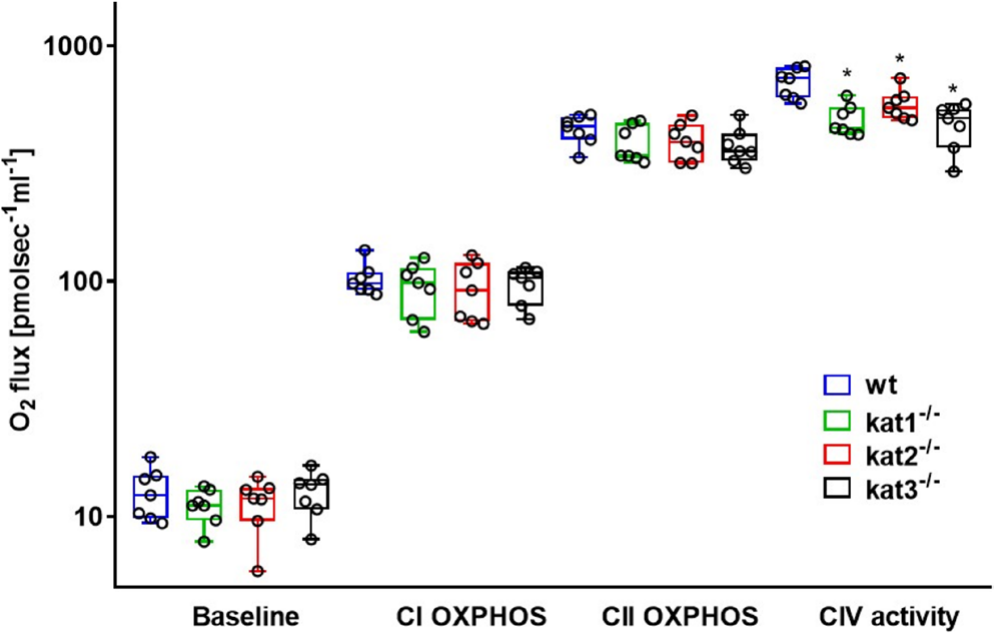
Baseline respiration, complex I‐, and complex II‐linked maximal capacities of oxidative phosphorylation (CI and CII OXPHOS) and complex IV (CIV) activities in wt (blue box), kat1^−/−^ (green box), kat2^−/−^ (red box), and kat3^−/−^ (black box) mouse liver (*n* = 7 homogenized liver samples from seven mice in each group). The plots demonstrate the median (horizontal line in the box) and the 25th (lower whisker) and 75th (upper whisker) percentiles. One‐way ANOVA with Holm–Sidak post hoc tests was conducted to determine the differences between the wt and KAT KO mouse strains. *p < 0.05 versus wt mice.

Among the non‐neuronal tissues, the liver was selected for mitochondrial O_2_ flux analysis, as illustrated in Figure 6. Although CIV‐stimulated respiration was attenuated in all KAT KO animals (*n* = 7, df_1_ = 3, df_2_ = 24, *F* = 9.499, *p* [kat1^−/−^] = 0.001, *p* [kat2^−/−^] = 0.048, *p* [kat3^−/−^] = 0.001, one‐way ANOVA), no statistically significant difference was found in hepatic baseline oxygen consumption rates (*n* = 7, df_1_ = 3, df_2_ = 24, *F* = 0.652, *p* = 0.589, one‐way ANOVA), and in ADP‐stimulated CI OXPHOS (*n* = 7, df_1_ = 3, df_2_ = 24, *F* = 0.255, *p* = 0.857, one‐way ANOVA) and CII OXPHOS (*n* = 7, df_1_ = 3, df_2_ = 24, *F* = 1.232, *p* = 0.320, one‐way ANOVA) states between the wt and KO mice.

### 3.5 Changes in Mitochondrial Membrane Potential (ΔΨmt)

**FIGURE 7:**
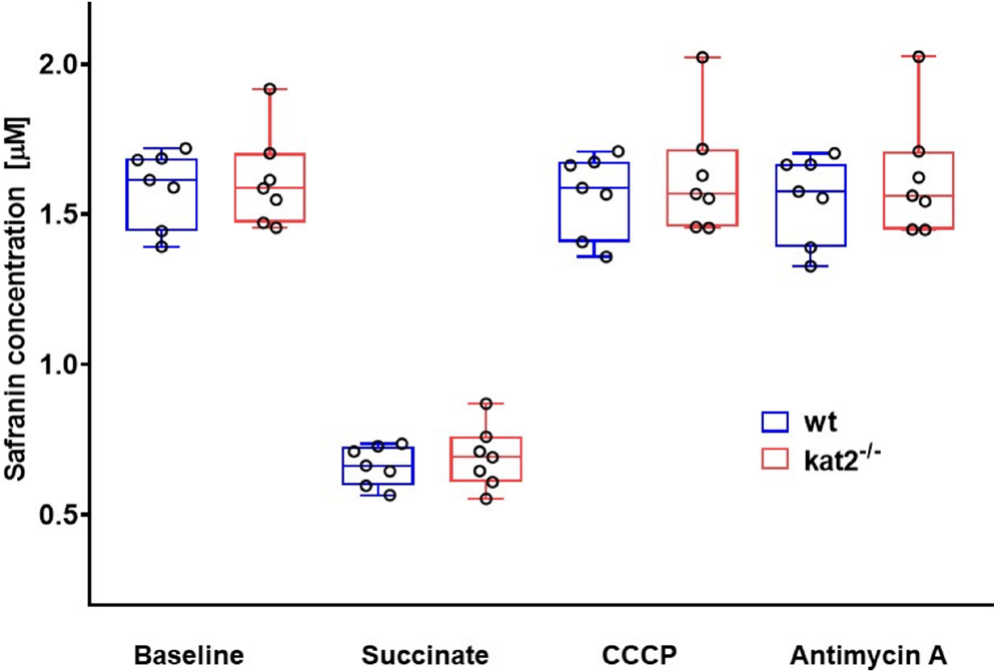
Changes in ΔΨmt. Baseline safranin concentrations, succinate‐induced hyperpolarization and CCCP‐induced depolarization (loss of ΔΨmt) were assessed in the cerebellum of wild type (blue box) and kat2^−/−^ (red box) mice (*n* = 7 homogenized cerebellum samples from seven mice in each group). The plots illustrate the median (horizontal line in the box) and the 25th (lower whisker) and 75th (upper whisker) percentiles. Student's *t* test was conducted to determine the difference between the wt and kat2^−/−^ mouse strains. *p < 0.05 versus wt mice.

After complex I inhibition and stabilization of fluorescence, baseline safranin concentrations were similar in wt and kat2^−/−^ mice, as illustrated in Figure 7 (*n* = 7, df = 12, *t* = −0.317, *p* = 0.757, *t* test). The addition of succinate resulted in an immediate decrease in fluorescent signal that was stabilized within 120 s (Figure 2). This substrate‐induced decrease (succinate‐induced hyperpolarization) was almost identical in wt and kat2^−/−^ groups (Figure 7; *n* = 7, df = 12, *t* = −0.595, *p* = 0.563, *t* test). Stepwise stimulation with an uncoupler resulted in depolarization (increases in safranin fluorescence) and loss of ΔΨmt (stabilization of fluorescence signal) at a critical CCCP concentration before ETS inhibition (antimycin A). We did not find marked differences in CCCP‐mediated ΔΨmt disruption between the groups (*n* = 7, df = 12, *t* = −0.0622, *p* = 0.504, *t* test).

## 4 Discussion

Pharmacological and genetic inhibition studies targeting CNS enzymes have garnered increasing attention for understanding dysfunctions in kynurenine pathway metabolism. Inhibitors of KAT II (e.g., quisqualate, BFF‐816) reduced cerebral KYNA levels, affected CNS function, and neurobiological processes related to schizophrenia and bipolar disorder (Guidetti et al. 1997; Wu et al. 2014). Similarly, a recent study by Szabó et al. (2025) revealed complex changes in Trp metabolism in newly developed kat2^−/−^ transgenic animals. The eight‐week‐old KO mice exhibited lower plasma KYN, 5‐hydroxyindolacetic acid, and indole‐3‐acetic acid concentrations. Enzyme activities of KATs, kynureninase, and monoamine oxidase/aldehyde dehydrogenase were reduced, while kynurenine 3‐monooxygenase activity was elevated. Additionally, markers of oxidative stress and excitotoxicity were markedly increased, and depression‐like behavior manifested in the kat2^−/−^ strain. On the other hand, Potter's group using 3‐week‐old juvenile KAT KO mice (FVB/N background) revealed an increase in extracellular glutamate levels, enhanced cognitive abilities, and synaptic plasticity due to lower KYNA content (Potter et al. 2010). These findings suggest that KATs play an essential role in the physiological functions of the CNS via the regulation of endogenous KYNA biosynthesis.

Mitochondrial dysfunction is a common denominator in the development of various neurodegenerative disorders (Bustamante‐Barrientos et al. 2023). Ongoing in vitro and in vivo studies highlight mitochondrial dysfunction, which is characterized by numerous molecular and cellular anomalies including calcium and iron homeostasis, ATP synthesis, and ROS production, among other factors. These multifactorial complexities of organelle dysfunction complicate their collective resolution by currently used experimental strategies. Although various preclinical studies revealed the mitoprotective effects of exogenous KYNA and KYNA analogues (Juhász et al. 2020; Poles et al. 2021), some effects of endogenous KYNA still remain elusive.

In this study, we first examined the mitochondrial consequences of CRISPR/Cas9‐mediated KO of the KYNA synthetizing KAT enzymes in mice. Our ultimate goal was to compare mitochondrial oxygen consumption associated with different electron transport chain complexes across various brain regions in newly created KAT KO animals. We found that KAT deficiency significantly affected mitochondrial oxygen consumption in different brain regions. Thus, ADP‐activated respiration, an indicator of energy production efficacy by OXPHOS, was decreased in the cerebellum, hippocampus (all three KAT KO) and striatum (kat2^−/−^ and kat3^−/−^), and these changes were accompanied by lower CIV activities.

**FIGURE 8:**
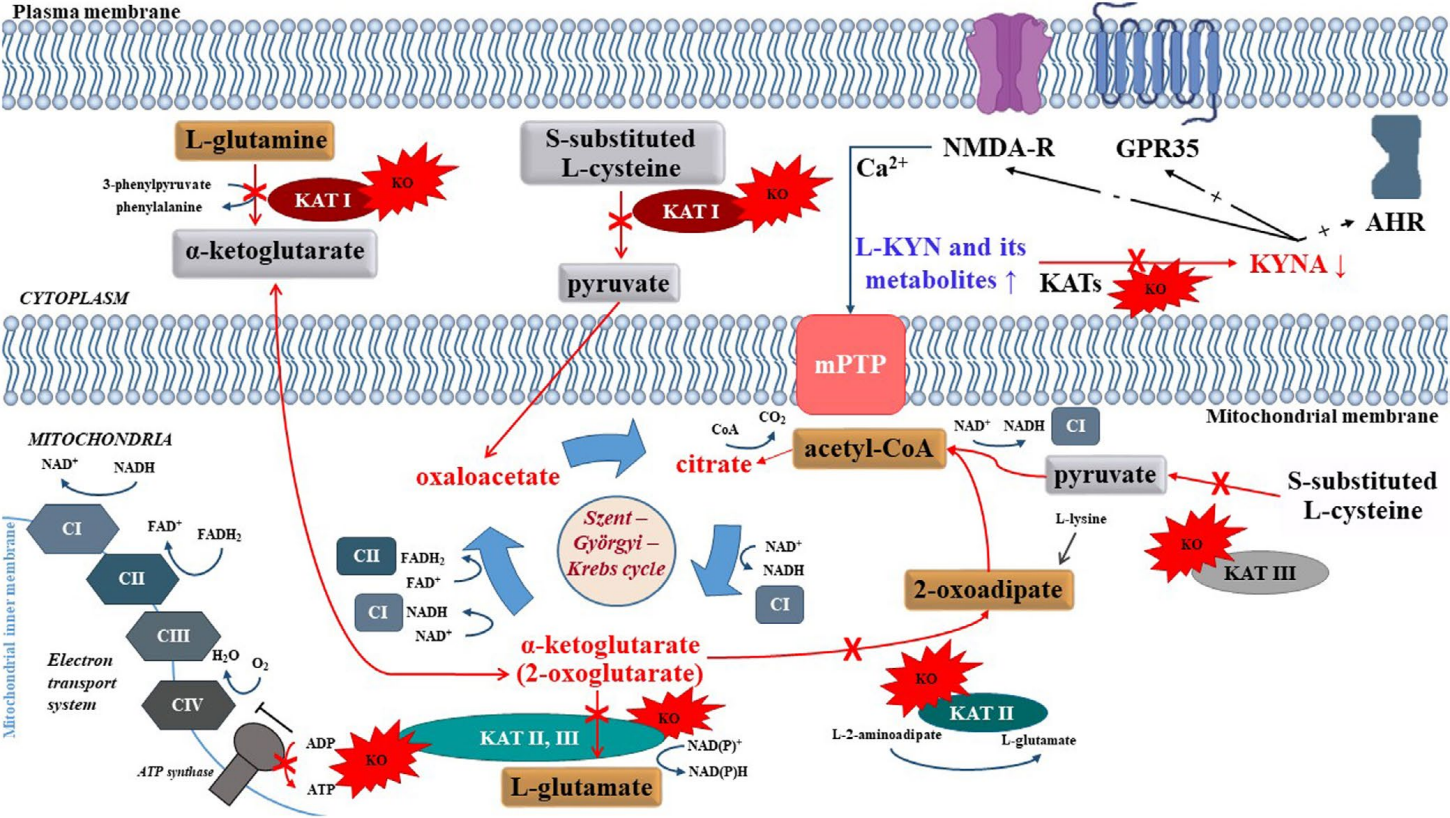
Possible mitochondrial effect of KAT KO. Reduced cerebral KYNA formation resulting from KAT KO may influence Ca^2+^ fluxes via NMDA‐R, energy utilization via GPR35, and mitochondrial homeostasis via AHR (KYNA‐dependent mechanisms). The concurrent disruption in the levels of L‐KYN and its further metabolites may further aggravate the decline in OXPHOS, potentially leading to diminished energy production. KATs are involved in the synthesis of pyruvate (KAT I, III), acetyl‐CoA (KAT II), and α‐ketoglutaric acid (KAT I; KYN pathway‐independent mechanisms). These metabolites can enter the Szent‐Györgyi‐Krebs (TCA) cycle and be used for NADH and FADH_2_ synthesis. NADH and FADH_2_, produced by the TCA cycle, play a crucial role in the electron transport system (ETS), to generate ATP via oxidative phosphorylation (OXPHOS). KAT KO may lead to a decrease in the level of reducing equivalents, eventually resulting in a decrease in mitochondrial substrate oxidation and ATP production. AHR, aryl hydrocarbon receptor; CI, respiratory complex I; CII, complex II of electron transport system;CoA, coenzyme A; FAD^+^, flavin adenine dinucleotide; GPR‐35, G protein‐coupled receptor 35; KAT I, II, III, IV, kynurenine aminotransferase I, II, III, IV; KYN, kynurenine; KYNA, kynurenic acid; mPTP: mitochondrial permeability transition pore; NAD(P)^+^, nicotinamide adenine dinucleotide phosphate; NAD^+^, nicotinamide adenine dinucleotide; NMDA‐R, N‐methyl‐d‐aspartate receptor.

We hypothesize that KAT KO‐induced decrease in mitochondrial respiration and lower ADP‐ATP conversion can be attributed to: (i) the reduction of cerebral KYNA formation and (ii) increased levels of L‐KYN and its downstream breakdown products (e.g., 3‐hydroxykynurenine, and 3‐hydroxyanthranilic acid) or (iii) KYN pathway independent mechanisms, including reactions linked to the Szent‐Györgyi‐Krebs cycle within mitochondria (Figure 8). The fundamental task of KAT isozymes is the synthesis of KYNA during the irreversible transamination of L‐KYN. It is likely that in all KAT KO mice, as a result of attenuated enzyme activity, the production of endogenous KYNA is reduced, affecting a wide range of cellular and subcellular processes.

For example, KYNA plays a role in fine‐tuning NMDA receptor (NMDA‐R) function since it is an endogenous antagonist of NMDA‐R. Reduced KYNA levels can affect receptor‐mediated signaling pathways, including Ca^2+^ entry into cells and mitochondria. Reduced inhibitory effects on NMDA‐Rs increase Ca^2+^ influx, which in turn affects TCA cycle enzymes (matrix dehydrogenases), electron transport chain function, antioxidant system, neurotransmitter clearance, and energy production in a concentration‐dependent manner (Denton 2009; Glancy and Balaban 2012; Nászai et al. 2019). High Ca^2+^ concentrations induce the opening of the mitochondrial permeability transition pore (mPTP) and increase inner membrane permeability that processes ultimately lead to apoptotic cell death (Kroemer et al. 1998; Bernardi et al. 2023; Baev et al. 2024).

Suppression of other KYNA‐ and receptor‐mediated mechanisms cannot be excluded, as KYNA also acts as an agonist for the aryl hydrocarbon receptor (AHR) and G protein‐coupled receptor 35 (GPR35) (Wirthgen et al. 2018). Energy utilization, including increased lipid metabolism and mitochondrial respiration, is modulated via GPR35 (Agudelo et al. 2018), while the maintenance of mitochondrial homeostasis, particularly through the regulation of the mitophagy receptor BCL2 interacting protein 3 is controlled via AHR (Heo et al. 2023).

Additionally, KATs not only catalyze the KYN‐KYNA conversion, but are also involved in the catalysis of other reactions linked to the Szent‐Györgyi‐Krebs cycle (Tanaka et al. 2022). Through these mechanisms, KATs may influence ATP synthesis in a KYNA‐ and receptor‐independent manner (Figure 8). In addition to the glycolytic pathway, pyruvate can be formed from l‐cysteine during amino acid metabolism, catalyzed by the KAT I enzyme in the cytoplasm and the KAT III enzyme in the mitochondrial matrix. If pyruvate does not enter the Szent‐Györgyi‐Krebs cycle, it is converted to acetyl‐CoA, while NADH is formed. The resulting NADH is a substrate of complex I, and NADH oxidation is coupled by the enzyme complex to the reduction of ubiquinone and the creation of a proton gradient, which is ultimately converted into ATP production at complex V (ATP synthase). Thus, it cannot be ruled out that the decreased mitochondrial respiration in KAT enzyme‐deficient mice is due to lower endogenous substrate availability or disturbances in TCA metabolite levels.

Moreover, KAT enzymes are involved in glutamate, lysine, and aspartate metabolism (Tanaka et al. 2022). The KAT II enzyme catalyzes the conversion of α‐ketoglutarate to 2‐oxoadipate, a precursor for acetyl‐CoA formation. It can be assumed that the α‐ketoglutarate‐2‐oxoadipate‐acetyl‐CoA conversion is inhibited due to the non‐functional KAT II enzyme in KO mice. Acetyl‐CoA is necessary for the first rate‐limiting step of the Szent‐Györgyi‐Krebs cycle, which converts oxaloacetate to citrate. In its absence, the amount of reducing equivalents (NADH and FADH_2_) decreases, leading to reduced OXPHOS capacity.

Mitochondrial membrane potential (Δψmt) is a key indicator of cell viability; dissipation of Δψmt is associated with cellular stress and apoptosis, and Δψmt disruption may promote mPTP‐mediated cell death. ETS and OXPHOS are controlled by the Δψmt and ATP, with ATP directly inhibiting cytochrome c oxidase (CIV), independent of Δψmt in living eukaryotic organisms (Kadenbach 2021; Ramzan et al. 2010, 2022). During safranin fluorescence measurements, we found no difference in succinate‐induced hyperpolarization or CCCP‐stimulated depolarization between wt and kat2^−/−^, suggesting that mitochondrial ATP synthesis regulation occurs independently of Δψmt. Since CIV activity decreased in all three examined KO strains, it is more likely that the reduced OXPHOS results from allosteric inhibition by the altered ATP/ADP ratio at CIV, rather than from Δψmt dissipation or mPTP opening.

ETS dysfunction has an impact on neuronal communication and neurotransmitter release. A study by Kilbride et al. (2011) has shown that high‐level inhibition of complexes III and IV is required to increase glutamate release from the nerve terminal and suggests that this mechanism may play an important role in neurodegeneration. Whether this mitochondria‐controlled neurotransmitter release occurs in KAT KO mice warrants further investigation.

One notable strength of this study is the use of tissue homogenates and the examination of three distinct brain regions for in‐depth mitochondrial measurements. Tissue homogenate offers several advantages over isolated mitochondria, including a quicker preparation, better preservation of tissue heterogeneity, and the need for only a small amount of tissue for functional mitochondrial studies (Nászai et al. 2019).

The study design has some limitations due to (I) its exclusive reliance on the High‐Resolution FluoRespirometry technique, (II) the absence of measurements for TCA cycle metabolites and reducing equivalents, and (III) the lack of comparison between KAT KO‐mediated mitochondrial changes and treatments using KAT enzyme inhibitors.

We plan to address this in future studies, including investigating KYNA and KYNA analogues (SZR‐72 and SZR‐104) therapy in KO animals. We hope that these new KO strains may serve as a valuable tool for preclinical research (despair‐based depression and post‐traumatic stress disorder), as KO mice exhibit complex metabolic, behavioral, and mitochondrial disturbances (Szabó et al. 2025). Alleviating mitochondrial dysfunction through the modulation of the Trp‐KYN pathway may be beneficial by maintaining energy metabolism in a variety of diseased conditions, such as Alzheimer's, Huntington's, Parkinson's, psychiatric diseases, and migraine.

We conclude that the deletion of the KAT genes significantly impairs mitochondrial respiration and ATP synthesis, potentially contributing to the development of various diseases. While the precise mechanisms remain unclear, evidence suggests that both KYNA‐dependent and KYNA‐independent processes may play a role in the observed mitochondrial dysfunction within the brain. This impairment could result from either a suppression of endogenous KYNA production or an imbalance in TCA cycle metabolites due to KAT KO. This study highlights the crucial role of KYNA in maintaining mitochondrial function and offers new perspectives on potential therapeutic targets for a range of neurological disorders. By modulating the KYN pathway, it may be possible to mitigate mitochondrial dysfunction and address the associated metabolic disturbances observed in numerous neuroinflammatory and neurodegenerative conditions. Overall, our findings suggest that targeting the KYN pathway could be a promising strategy to improve mitochondrial health and function, potentially leading to novel treatments for these challenging diseases.

## Author Contributions

**László Juhász:** conceptualization, data curation, writing – original draft, investigation, methodology, writing – review and editing, funding acquisition. **Krisztina Spisák:** investigation, data curation, writing – original draft, methodology. **Boglárka Zsuzsa Szolnoki:** investigation, methodology, data curation. **Anna Nászai:** investigation, methodology, data curation. **Ágnes Szabó:** investigation, writing – original draft, methodology. **Attila Rutai:** investigation, writing – original draft, methodology. **Szabolcs Péter Tallósy:** investigation, data curation, writing – original draft, methodology. **Andrea Szabó:** writing – review and editing. **József Toldi:** conceptualization, writing – review and editing. **Masaru Tanaka:** conceptualization, writing – original draft, writing – review and editing. **Keiko Takeda:** investigation, methodology. **Kinuyo Ozaki:** methodology, investigation. **Hiromi Inoue:** investigation, methodology. **Sayo Yamamoto:** investigation, methodology. **Etsuro Ono:** supervision, writing – review and editing. **Mihály Boros:** supervision, writing – review and editing. **József Kaszaki:** supervision, writing – review and editing. **László Vécsei:** conceptualization, writing – review and editing, writing – original draft, supervision, project administration, funding acquisition.

## Conflicts of Interest

The authors declare no conflicts of interest.

### Peer Review

The peer review history for this article is available at https://www.webofscience.com/api/gateway/wos/peer‐review/10.1111/jnc.70075.

## Supporting information


Data S1.

## Data Availability

The data that support the findings of this study are available from the corresponding author upon reasonable request.

## References

[jnc70075-bib-0001] Agudelo, L. Z. , D. M. S. Ferreira , I. Cervenka , et al. 2018. “Kynurenic Acid and Gpr35 Regulate Adipose Tissue Energy Homeostasis and Inflammation.” Cell Metabolism 27, no. 2: 378–392.e5. 10.1016/j.cmet.2018.01.004. 29414686

[jnc70075-bib-0002] Baev, A. Y. , A. Y. Vinokurov , E. V. Potapova , A. V. Dunaev , P. R. Angelova , and A. Y. Abramov . 2024. “Mitochondrial Permeability Transition, Cell Death and Neurodegeneration.” Cells 13, no. 7: 648. 10.3390/cells13070648. 38607087 PMC11011324

[jnc70075-bib-0003] Balla, Z. , E. S. Kormányos , B. Kui , et al. 2021. “Kynurenic Acid and Its Analogue SZR‐72 Ameliorate the Severity of Experimental Acute Necrotizing Pancreatitis.” Frontiers in Immunology 12: 702764. 10.3389/fimmu.2021.702764. 34745090 PMC8567016

[jnc70075-bib-0004] Baran, H. , K. Staniek , M. Bertignol‐Spörr , M. Attam , C. Kronsteiner , and B. Kepplinger . 2016. “Effects of Various Kynurenine Metabolites on Respiratory Parameters of Rat Brain, Liver and Heart Mitochondria.” International Journal of Tryptophan Research: IJTR 9: 17–29. 10.4137/IJTR.S37973. 27226722 PMC4872644

[jnc70075-bib-0005] Bernardi, P. , C. Gerle , A. P. Halestrap , et al. 2023. “Identity, Structure, and Function of the Mitochondrial Permeability Transition Pore: Controversies, Consensus, Recent Advances, and Future Directions.” Cell Death and Differentiation 30, no. 8: 1869–1885. 10.1038/s41418-023-01187-0. 37460667 PMC10406888

[jnc70075-bib-0006] Bustamante‐Barrientos, F. A. , N. Luque‐Campos , M. J. Araya , et al. 2023. “Mitochondrial Dysfunction in Neurodegenerative Disorders: Potential Therapeutic Application of Mitochondrial Transfer to Central Nervous System‐Residing Cells.” Journal of Translational Medicine 21, no. 1: 613. 10.1186/s12967-023-04493-w. 37689642 PMC10493034

[jnc70075-bib-0007] Carrì, M. T. , C. Valle , F. Bozzo , and M. Cozzolino . 2015. “Oxidative Stress and Mitochondrial Damage: Importance in Non‐SOD1 ALS.” Frontiers in Cellular Neuroscience 9: 41. 10.3389/fncel.2015.00041. 25741238 PMC4330888

[jnc70075-bib-0008] Denton, R. M. 2009. “Regulation of Mitochondrial Dehydrogenases by Calcium Ions.” Biochimica et Biophysica Acta 1787, no. 11: 1309–1316. 10.1016/j.bbabio.2009.01.005. 19413950

[jnc70075-bib-0009] Érces, D. , G. Varga , B. Fazekas , et al. 2012. “N‐Methyl‐D‐Aspartate Receptor Antagonist Therapy Suppresses Colon Motility and Inflammatory Activation Six Days After the Onset of Experimental Colitis in Rats.” European Journal of Pharmacology 691, no. 1–3: 225–234. 10.1016/j.ejphar.2012.06.044. 22796676

[jnc70075-bib-0010] Fujigaki, H. , Y. Yamamoto , and K. Saito . 2017. “L‐Tryptophan‐Kynurenine Pathway Enzymes Are Therapeutic Target for Neuropsychiatric Diseases: Focus on Cell Type Differences.” Neuropharmacology 112, no. Pt B: 264–274. 10.1016/j.neuropharm.2016.01.011. 26767951

[jnc70075-bib-0011] Glancy, B. , and R. S. Balaban . 2012. “Role of Mitochondrial Ca^2+^ in the Regulation of Cellular Energetics.” Biochemistry 51, no. 14: 2959–2973. 10.1021/bi2018909. 22443365 PMC3332087

[jnc70075-bib-0012] Golpich, M. , E. Amini , Z. Mohamed , R. Azman Ali , N. Mohamed Ibrahim , and A. Ahmadiani . 2017. “Mitochondrial Dysfunction and Biogenesis in Neurodegenerative Diseases: Pathogenesis and Treatment.” CNS Neuroscience & Therapeutics 23, no. 1: 5–22. 10.1111/cns.12655. 27873462 PMC6492703

[jnc70075-bib-0013] Guidetti, P. , E. Okuno , and R. Schwarcz . 1997. “Characterization of Rat Brain Kynurenine Aminotransferases I and II.” Journal of Neuroscience Research 50, no. 3: 457–465. 10.1002/(SICI)1097-4547(19971101)50:3<457::AID-JNR12>3.0.CO;2-3. 9364331

[jnc70075-bib-0014] Han, Q. , T. Cai , D. A. Tagle , and J. Li . 2010. “Structure, Expression, and Function of Kynurenine Aminotransferases in Human and Rodent Brains.” Cellular and Molecular Life Sciences: CMLS 67, no. 3: 353–368. 10.1007/s00018-009-0166-4. 19826765 PMC2867614

[jnc70075-bib-0015] Heo, M. J. , J. H. Suh , S. H. Lee , et al. 2023. “Aryl Hydrocarbon Receptor Maintains Hepatic Mitochondrial Homeostasis in Mice.” Molecular Metabolism 72: 101717. 10.1016/j.molmet.2023.101717. 37004989 PMC10106517

[jnc70075-bib-0016] Johansson, A. S. , B. Owe‐Larsson , L. Asp , et al. 2013. “Activation of Kynurenine Pathway in Ex Vivo Fibroblasts From Patients With Bipolar Disorder or Schizophrenia: Cytokine Challenge Increases Production of 3‐Hydroxykynurenine.” Journal of Psychiatric Research 47, no. 11: 1815–1823. 10.1016/j.jpsychires.2013.08.008. 24012176

[jnc70075-bib-0017] Juhász, L. , A. Rutai , R. Fejes , et al. 2020. “Divergent Effects of the N‐Methyl‐D‐Aspartate Receptor Antagonist Kynurenic Acid and the Synthetic Analog SZR‐72 on Microcirculatory and Mitochondrial Dysfunction in Experimental Sepsis.” Frontiers in Medicine 7: 566582. 10.3389/fmed.2020.566582. 33330526 PMC7729001

[jnc70075-bib-0018] Kadenbach, B. 2021. “Complex IV–The Regulatory Center of Mitochondrial Oxidative Phosphorylation.” Mitochondrion 58: 296–302. 10.1016/j.mito.2020.10.004. 33069909

[jnc70075-bib-0019] Kawai, S. , Y. Takagi , S. Kaneko , and T. Kurosawa . 2011. “Effect of Three Types of Mixed Anesthetic Agents Alternate to Ketamine in Mice.” Experimental Animals 60, no. 5: 481–487. 10.1538/expanim.60.481. 22041285

[jnc70075-bib-0020] Kilbride, S. M. , S. A. Gluchowska , J. E. Telford , C. O'Sullivan , and G. P. Davey . 2011. “High‐Level Inhibition of Mitochondrial Complexes III and IV Is Required to Increase Glutamate Release From the Nerve Terminal.” Molecular Neurodegeneration 6, no. 1: 53. 10.1186/1750-1326-6-53. 21791084 PMC3169489

[jnc70075-bib-0021] Kroemer, G. , B. Dallaporta , and M. Resche‐Rigon . 1998. “The Mitochondrial Death/Life Regulator in Apoptosis and Necrosis.” Annual Review of Physiology 60: 619–642. 10.1146/annurev.physiol.60.1.619. 9558479

[jnc70075-bib-0022] Krumschnabel, G. , A. Eigentler , M. Fasching , and E. Gnaiger . 2014. “Use of Safranin for the Assessment of Mitochondrial Membrane Potential by High‐Resolution Respirometry and Fluorometry.” Methods in Enzymology 542: 163–181. 10.1016/B978-0-12-416618-9.00009-1. 24862266

[jnc70075-bib-0023] Liu, H. , L. Ding , H. Zhang , et al. 2018. “The Metabolic Factor Kynurenic Acid of Kynurenine Pathway Predicts Major Depressive Disorder.” Frontiers in Psychiatry 9: 552. 10.3389/fpsyt.2018.00552. 30510519 PMC6252326

[jnc70075-bib-0024] Martin, L. J. 2010. “Mitochondrial and Cell Death Mechanisms in Neurodegenerative Diseases.” Pharmaceuticals 3, no. 4: 839–915. 10.3390/ph3040839. 21258649 PMC3023298

[jnc70075-bib-0025] Mithaiwala, M. N. , D. Santana‐Coelho , G. A. Porter , and J. C. O'Connor . 2021. “Neuroinflammation and the Kynurenine Pathway in CNS Disease: Molecular Mechanisms and Therapeutic Implications.” Cells 10, no. 6: 1548. 10.3390/cells10061548. 34205235 PMC8235708

[jnc70075-bib-0026] Naito, Y. , K. Hino , H. Bono , and K. Ui‐Tei . 2015. “CRISPRdirect: Software for Designing CRISPR/Cas Guide RNA With Reduced Off‐Target Sites.” Bioinformatics 31, no. 7: 1120–1123. 10.1093/bioinformatics/btu743. 25414360 PMC4382898

[jnc70075-bib-0027] Nászai, A. , E. Terhes , J. Kaszaki , M. Boros , and L. Juhász . 2019. “Ca^(2+)^N It be Measured? Detection of Extramitochondrial Calcium Movement With High‐Resolution FluoRespirometry.” Scientific Reports 9, no. 1: 19229. 10.1038/s41598-019-55618-5. 31848391 PMC6917783

[jnc70075-bib-0028] Norat, P. , S. Soldozy , J. D. Sokolowski , et al. 2020. “Mitochondrial Dysfunction in Neurological Disorders: Exploring Mitochondrial Transplantation.” Npj Regenerative Medicine 5, no. 1: 22. 10.1038/s41536-020-00107-x. 33298971 PMC7683736

[jnc70075-bib-0029] Palzkill, V. R. , T. Thome , A. L. Murillo , R. B. Khattri , and T. E. Ryan . 2022. “Increasing Plasma L‐Kynurenine Impairs Mitochondrial Oxidative Phosphorylation Prior to the Development of Atrophy in Murine Skeletal Muscle: A Pilot Study.” Frontiers in Physiology 13: 992413. 10.3389/fphys.2022.992413. 36246103 PMC9562971

[jnc70075-bib-0030] Poles, M. Z. , A. Nászai , L. Gulácsi , et al. 2021. “Kynurenic Acid and Its Synthetic Derivatives Protect Against Sepsis‐Associated Neutrophil Activation and Brain Mitochondrial Dysfunction in Rats.” Frontiers in Immunology 12: 717157. 10.3389/fimmu.2021.717157. 34475875 PMC8406694

[jnc70075-bib-0031] Potter, M. C. , G. I. Elmer , R. Bergeron , et al. 2010. “Reduction of Endogenous Kynurenic Acid Formation Enhances Extracellular Glutamate, Hippocampal Plasticity, and Cognitive Behavior.” Neuropsychopharmacology 35, no. 8: 1734–1742. 10.1038/npp.2010.39. 20336058 PMC3055476

[jnc70075-bib-0032] Ramzan, R. , A. M. Dolga , S. Michels , et al. 2022. “Cytochrome c Oxidase Inhibition by ATP Decreases Mitochondrial ROS Production.” Cells 11, no. 6: 992. 10.3390/cells11060992. 35326443 PMC8946758

[jnc70075-bib-0033] Ramzan, R. , K. Staniek , B. Kadenbach , and S. Vogt . 2010. “Mitochondrial Respiration and Membrane Potential Are Regulated by the Allosteric ATP‐Inhibition of Cytochrome c Oxidase.” Biochimica et Biophysica Acta 1797, no. 9: 1672–1680. 10.1016/j.bbabio.2010.06.005. 20599681

[jnc70075-bib-0034] Sas, K. , E. Szabó , and L. Vécsei . 2018. “Mitochondria, Oxidative Stress and the Kynurenine System, With a Focus on Ageing and Neuroprotection.” Molecules 23, no. 1: 191. 10.3390/molecules23010191. 29342113 PMC6017505

[jnc70075-bib-0035] Savitz, J. 2020. “The Kynurenine Pathway: A Finger in Every Pie.” Molecular Psychiatry 25, no. 1: 131–147. 10.1038/s41380-019-0414-4. 30980044 PMC6790159

[jnc70075-bib-0036] Seo, S. K. , and B. Kwon . 2023. “Immune Regulation Through Tryptophan Metabolism.” Experimental & Molecular Medicine 55, no. 7: 1371–1379. 10.1038/s12276-023-01028-7. 37394584 PMC10394086

[jnc70075-bib-0037] Song, T. , X. Song , C. Zhu , et al. 2021. “Mitochondrial Dysfunction, Oxidative Stress, Neuroinflammation, and Metabolic Alterations in the Progression of Alzheimer's Disease: A Meta‐Analysis of In Vivo Magnetic Resonance Spectroscopy Studies.” Ageing Research Reviews 72: 101503. 10.1016/j.arr.2021.101503. 34751136 PMC8662951

[jnc70075-bib-0038] Szabó, Á. , Z. Galla , E. Spekker , et al. 2025. “Oxidative and Excitatory Neurotoxic Stresses in CRISPR/Cas9‐Induced Kynurenine Aminotransferase Knockout Mice: A Novel Model for Despair‐Based Depression and Post‐Traumatic Stress Disorder.” Frontiers in Bioscience 30, no. 1: 25706. 10.31083/FBL25706. 39862084

[jnc70075-bib-0039] Tanaka, M. , Z. Bohár , D. Martos , G. Telegdy , and L. Vécsei . 2020. “Antidepressant‐Like Effects of Kynurenic Acid in a Modified Forced Swim Test.” Pharmacological Reports: PR 72, no. 2: 449–455. 10.1007/s43440-020-00067-5. 32162182

[jnc70075-bib-0040] Tanaka, M. , Á. Szabó , E. Spekker , H. Polyák , F. Tóth , and L. Vécsei . 2022. “Mitochondrial Impairment: A Common Motif in Neuropsychiatric Presentation? The Link to the Tryptophan‐Kynurenine Metabolic System.” Cells 11, no. 16: 2607. 10.3390/cells11162607. 36010683 PMC9406499

[jnc70075-bib-0041] Tanaka, M. , F. Tóth , H. Polyák , Á. Szabó , Y. Mándi , and L. Vécsei . 2021. “Immune Influencers in Action: Metabolites and Enzymes of the Tryptophan‐Kynurenine Metabolic Pathway.” Biomedicine 9, no. 7: 734. 10.3390/biomedicines9070734. PMC8301407 34202246

[jnc70075-bib-0042] Varga, G. , D. Erces , B. Fazekas , et al. 2010. “N‐Methyl‐D‐Aspartate Receptor Antagonism Decreases Motility and Inflammatory Activation in the Early Phase of Acute Experimental Colitis in the Rat.” Neurogastroenterology and Motility 22, no. 2: e217–e268. 10.1111/j.1365-2982.2009.01390.x. 19735360

[jnc70075-bib-0043] Vécsei, L. , L. Szalárdy , F. Fülöp , and J. Toldi . 2013. “Kynurenines in the CNS: Recent Advances and New Questions.” Nature Reviews. Drug Discovery 12, no. 1: 64–82. 10.1038/nrd3793. 23237916

[jnc70075-bib-0044] Wirthgen, E. , A. Hoeflich , A. Rebl , and J. Günther . 2018. “Kynurenic Acid: The Janus‐Faced Role of an Immunomodulatory Tryptophan Metabolite and Its Link to Pathological Conditions.” Frontiers in Immunology 8: 1957. 10.3389/fimmu.2017.01957. 29379504 PMC5770815

[jnc70075-bib-0045] Wu, H. Q. , M. Okuyama , Y. Kajii , A. Pocivavsek , J. P. Bruno , and R. Schwarcz . 2014. “Targeting Kynurenine Aminotransferase II in Psychiatric Diseases: Promising Effects of an Orally Active Enzyme Inhibitor.” Schizophrenia Bulletin 40, no. Suppl 2: S152–S158. 10.1093/schbul/sbt157. 24562494 PMC3934402

