# Supplementary material for: The Power Struggle: Kynurenine Pathway Enzyme Knockouts and Brain Mitochondrial Respiration
Source: J Neurochem. 2025 May 2;169(5):e70075. doi: 10.1111/jnc.70075 (PMC12048769; doi:10.1111/jnc.70075)
Supplement: Supplementary file 1 — Data S1. [file JNC-169-0-s001.pdf]

The Power Struggle: Kynurenine Pathway Enzyme Knockouts and Brain Mitochondrial Respiration

László Juhász[1], Krisztina Spisák[1], Boglárka Zsuzsa Szolnoki[1], Anna Nászai[1], Ágnes Szabó[2,4], Attila Rutai[1], Szabolcs Péter Tallósy[1], Andrea Szabó[1], József Toldi[3], Masaru Tanaka[4], Keiko Takeda[5], Kinuyo Ozaki[6], Hiromi Inoue[6], Sayo Yamamoto[6], Etsuro Ono[5,6], Mihály Boros[1], József Kaszaki[1], László Vécsei[2,4]

[1]*Institute of Surgical Research, University of Szeged, Albert Szent-Györgyi Medical School, Szeged, Hungary*

[2]*Department of Neurology, University of Szeged, Albert Szent-Györgyi Medical School, Szeged, Hungary*

[3]*Department of Physiology, Anatomy and Neuroscience, University of Szeged, Szeged, Hungary*

[4]*HUN-REN-SZTE Neuroscience Research Group, Hungarian Research Network, University of Szeged (HUN-REN-SZTE), Danube Neuroscience Research Laboratory, Szeged, Hungary*

[5]*Department of Biomedicine, Graduate School of Medical Sciences, Kyushu University, Fukuoka, Japan*

[6]*Center of Biomedical Research, Research Center for Human Disease Modeling, Graduate School of Medical Sciences, Kyushu University, Fukuoka, Japan*

Corresponding author: László Juhász
University of Szeged, Albert Szent-Györgyi Medical School, Institute of Surgical Research, Szeged, Hungary
E-mail: juhasz.laszlo.1@med.u-szeged.hu
H-6720 Szőkefalvi-Nagy Béla utca 6, Szeged, Hungary
Phone: +36 62 545 103
Mobile:+36 20 417 1294

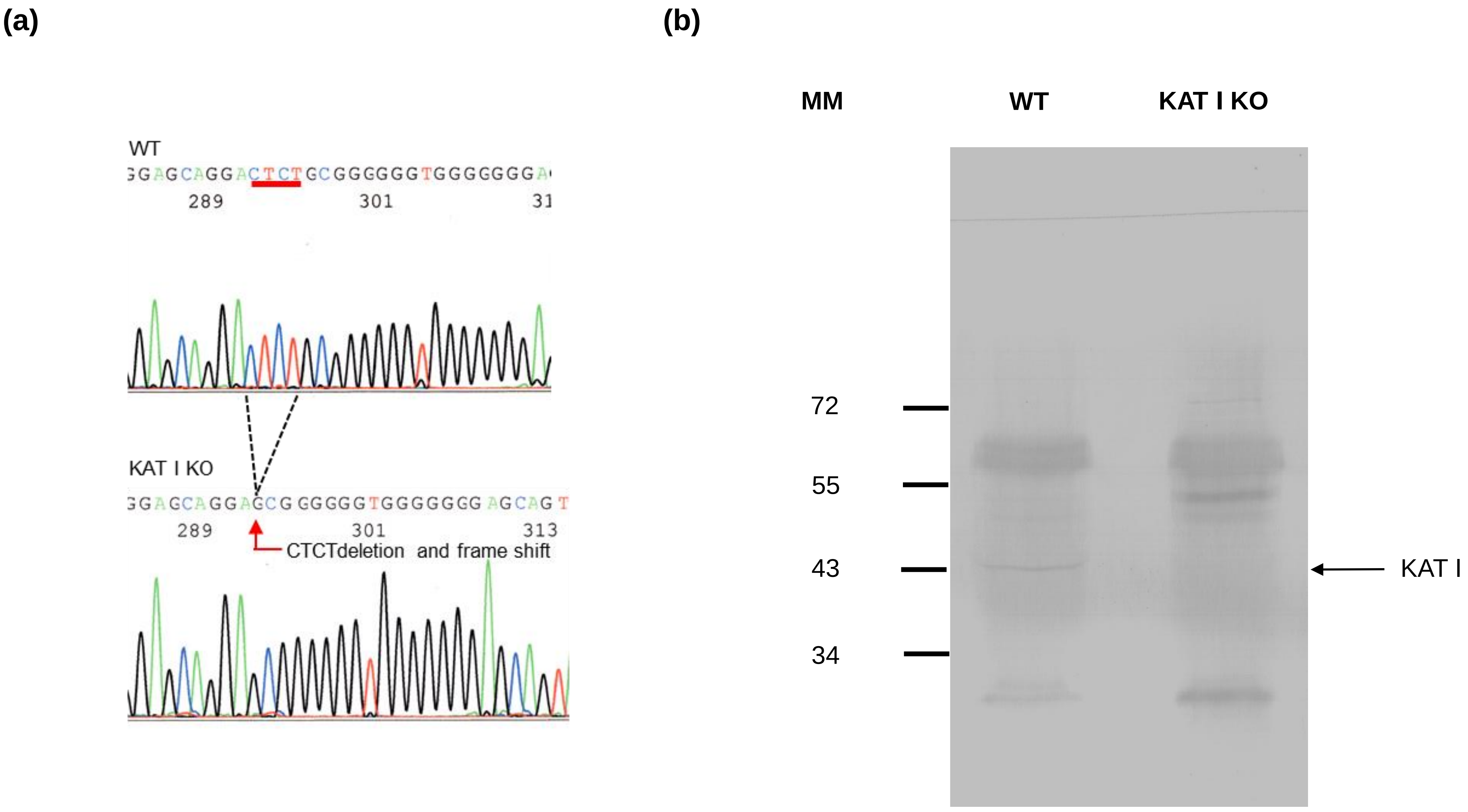

Supplementary Figure 1. CRISPR/Cas9-mediated gene KO of KAT I (a) and Western blot analysis of KAT I in liver lysates (b)

(a)

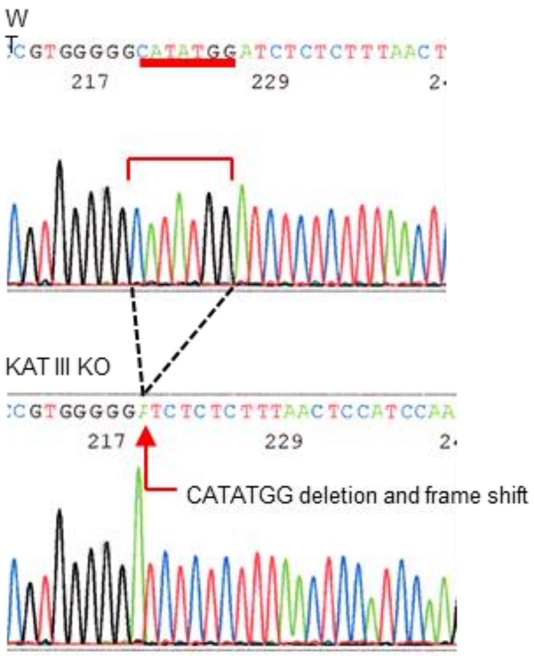

(b)

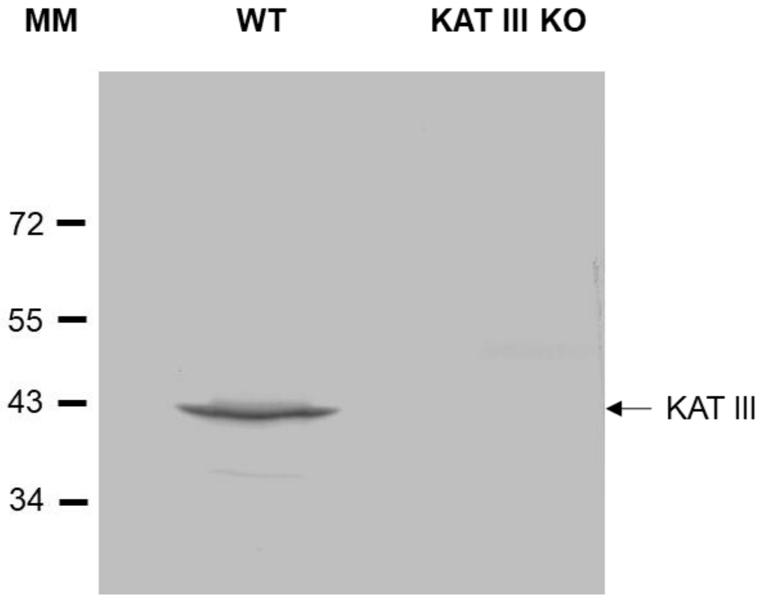

Supplementary Figure 2. CRISPR/Cas9-mediated gene KO of KAT III (a) and Western blot analysis of KAT III in liver lysates (b)
